# Clinical significance of serum bilirubin in primary Sjögren syndrome patients

**DOI:** 10.1002/jcla.23090

**Published:** 2019-11-06

**Authors:** Zunni Zhang, Qisheng Su, Liqian Zhang, Zheng Yang, Yuling Qiu, Wuning Mo

**Affiliations:** ^1^ Department of Clinical Laboratory First Affiliated Hospital of Guangxi Medical University Nanning China

**Keywords:** bilirubin, biomarker, inflammation, primary Sjögren syndrome

## Abstract

**Objective:**

The purpose of our research was to demonstrate the clinical significance of serum bilirubin in primary Sjögren syndrome patients (pSS).

**Patients and methods:**

A total of 116 patients with primary Sjögren syndrome and 138 matched individuals were included in our study. The laboratory parameters of patients with pSS and healthy controls were retrospectively analyzed.

**Results:**

Serum total bilirubin, direct bilirubin, and indirect bilirubin were significantly reduced (*P* < .001, *P* = .001, *P* < .001) while ESR was significantly increased (*P* < .001) in patients with pSS when compared with healthy checkup individuals. Statistically, the AUC in patients with pSS is as follows: TBIL = 0.77, *P* < .001, cutoff value = 7.96; DBIL = 0.617, *P* = .001 cutoff value = 2.2; and IBIL = 0.786, *P* < .001 cutoff value = 4.5. Furthermore, our study revealed that TBIL, DBIL, and IBIL were significantly negativity related to ESR (*r* = −.406, *P* < .001; *r* = −.206, *P* = .026; *r* = −.429, *P* < .001). Interestingly, multiple linear regression analysis showed that when adjusted for sex, age, ALT, and AST, the levels of TBIL, DBIL, and IBIL in patients with pSS were independently correlated with ESR.

**Conclusions:**

This study found that the levels of serum bilirubin were reduced and the inflammatory marker was elevated in patients with pSS. Additionally, serum bilirubin was negatively related with ESR and TBIL, DBIL, and IBIL can be used in the clinical diagnosis and follow‐up visits of the patients with pSS.

**Abbreviations**
AUC: the area under the ROC curve
DBIL: direct bilirubin
ESR: erythrocyte sedimentation rate
IBIL: indirect bilirubin
OS: oxidative stress
PM: polymyositis
pSS: primary Sjögren syndrome
RA: rheumatoid arthritis
ROC: receiver operating characteristic curve
SLE: systemic lupus erythematosus
TBIL: total bilirubin

## 1 INTRODUCTION

Primary Sjögren syndrome（pSS）is an autoimmune and chronic inflammatory disease that can invade the exocrine gland of the whole body, especially the saliva and lacrimal gland.^1^, ^2^ The characteristics of pSS are dental caries and recurrent mumps, especially dry mouth and eyes. Besides, it can also involve other organs to cause a variety of clinical damage.^3^ It is a common disease with the global incidence range from 0.05% to 0.5%, but most frequent in women.^4^ Until now, although years of efforts have demonstrated that the development of pSS is related to genetic, environmental, and immunological basis, the exact cause is still unknown.^5^ Previous studies had showed that pSS is related to some inflammatory cytokines such as interleukin‐10 (IL‐10), interleukin‐17(IL‐17), interferon‐*γ*, and interleukin‐37 (IL‐37),^1^, ^6^, ^7^, ^8^ but they are too expensive to routine inspection.

As the final decomposition product of heme metabolism, functions of serum bilirubin not only in the endogenous strong antioxidant but also in anti‐inflammation and immunosuppression provide significant protection for many diseases. Previous studies have discovered that the concentration of bilirubin was reduced in migraine, cardiovascular disease, and Crohn's disease patients.^9^, ^10^, ^11^ Furthermore, serum bilirubin also plays a crucial role in autoimmune diseases, such as rheumatoid arthritis (RA), polymyositis (PM), and systemic lupus erythematosus (SLE).^12^, ^13^, ^14^ Despite the fact that a variety of studies have demonstrated that the levels of bilirubin were related to inflammatory disease but there still no study shows the correlation between pSS and bilirubin, this study investigated the relation between the levels of bilirubin and patients with pSS.

## 2 PATIENTS AND METHODS

From June 2015 to June 2019, 116 patients with pSS who diagnosed in the First Affiliated Hospital of Guangxi Medical University (Guangxi, China) were included in our study. Patients with pSS were diagnosed according to international standards.^15^ Additionally, 138 sex and age‐matched individuals who went to our hospital for healthy checkup at the same time were served as healthy controls. Patients with obesity, smoking, acute or chronic inflammatory diseases, cardiovascular disease, concomitant with other autoimmune diseases, anti‐inflammatory drugs and painkillers treatment in the recent month, and liver damage caused by diseases were ruled out our study.

Fasting blood collected from 116 patients with pSS before any treatment and 138 healthy controls. Serum total bilirubin (TBIL), direct bilirubin (DBIL), indirect bilirubin (IBIL), alanine aminotransferase (ALT), and aspartate aminotransferase (AST) were monitored by automatic biochemical Analyser 7600‐120 (Hitachi High Technologies, Japan). Moreover, automatic Analyser Minitor‐100 (Electa Lab Srl; Forli, Italy) was used to detect erythrocyte sedimentation rate (ESR). The Ethics Committee of the First Affiliated Hospital of Guangxi Medical University gave permission to this research.

### 2.1 Statistical analysis

The software of Statistical Product and Service Solutions (SPSS, version 24) was used for statistical analysis. We applied the Kolmogorov‐Smirnov test to distinguish the normality of the data, and *P* > .1 was regarded as normal distribution data. Normal distribution data were compared by independent Student's *t* test and described by means and standard deviations, if not, analyzed by Mann‐Whitney *U* test and represented by median and quartile spacing. The correlation analysis between two variables was performed by the spearman approach. The receiver operating characteristic curve (ROC) was applied to calculate the diagnostic value of TBIL, DBIL, and IBIL in patients with PSS. Additionally, multiple linear regression used ESR as the dependent variable, adjusted the effects of gender, age, ALT, and AST, and evaluated the relationship between TBIL, DBIL, IBIL, and ESR in patients with pSS.

## 3 RESULTS

The characteristics of patients with pSS and healthy individuals are shown in Table 1. Sex and age have no statistically significant differences between patients with pSS and healthy individuals. TBIL, DBIL, and IBIL were reduced in patients with pSS significantly (6.45, IQR: 4.92‐9.31 µmol/L; 2.17, IQR: 1.51‐3.29 µmol/L; 4.17, IQR: 3.03‐6.4 µmol/L) when compared with healthy controls (10.75, IQR: 8.88‐13 µmol/L; 2.8, IQR: 2.1‐3.63 µmol/L; 7.7, IQR: 6.1‐10 µmol/L) with *P* < .001, *P* = .001, *P* < .001 independently. Besides, ESR in patients with pSS was higher (38, IQR: 21‐57 mm/h) than healthy controls, significantly (13, IQR: 7‐19). Interestingly, ALT and AST between patients with pSS and healthy controls have no statistically significant difference with *P* = .071, *P* = .358, independently.

**Table 1:** Laboratory data of the primary Sjogren's syndrome patients and healthy controls

	pSS patients (n = 116)	Healthy controls (n = 138)	*P*‐value
Gender (male/female)	13/103	17/121	.785
Age (y)	47.05 ± 15.51	47.94 ± 15.19	.648
ESR (mm/h)	38 (21‐57)	13 (7‐19)	<.001
TBIL (µmol/L)	6.45 (4.92‐9.31)	10.75 (8.88‐13)	<.001
DBIL (µmol/L)	2.17 (1.51‐3.29)	2.8 (2.1‐3.63)	.001
IBIL (µmol/L)	4.17 (3.03‐6.4)	7.7 (6.1‐10)	<.001
ALT (U/L)	17 (12.25‐25)	16 (11‐22.5)	.071
AST (U/L)	20.5 (17‐29)	20 (17‐24.5)	.358

**Figure 1:**
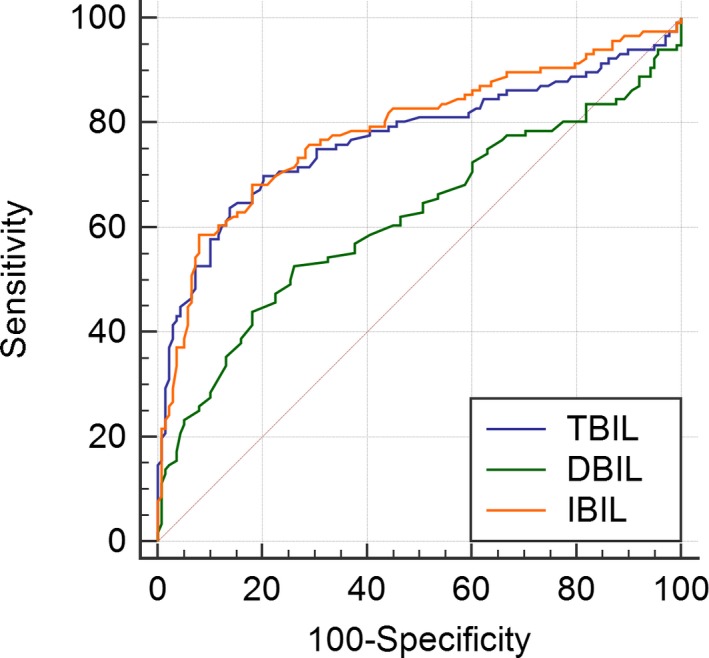
The roc curve of TBIL DBIL and IBIL

As show in Figure 1, receiver operating characteristic curve (ROC) revealed the diagnose value of TBIL (AUC = 0.77, specificity = 86.2%, sensitivity = 63.8%, cutoff value = 7.96, 95%CI = 0.713‐0.82, *P* < .001), DBIL (AUC = 0.617, specificity = 73.9%, sensitivity = 52.6%, cutoff value = 2.2, 95%CI = 0.555‐0.677, *P* = .001), and IBIL (AUC = 0.786, specificity = 92%, sensitivity = 58.6%, cutoff value = 4.5, 95%CI = 0.73‐0.835, *P* < .001) in patients with pSS.

**Figure 2:**
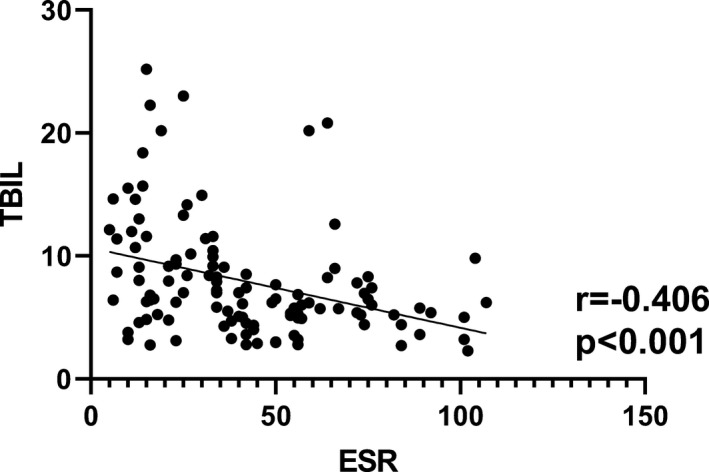
Correlation analysis between ESR and TBIL

**Figure 3:**
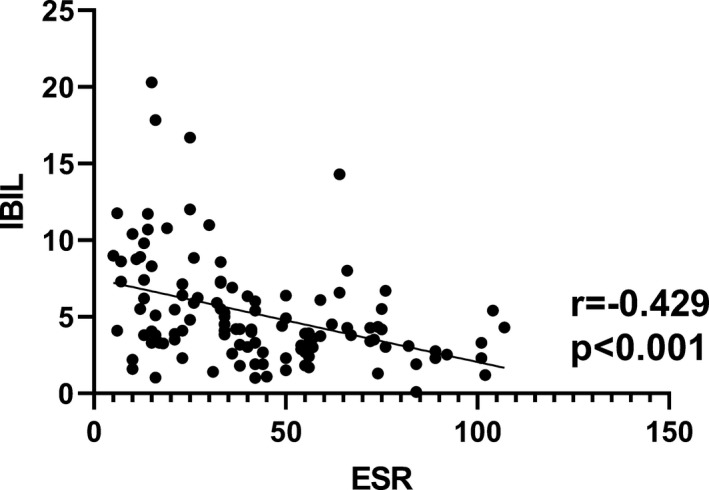
Correlation analysis between ESR and IBIL

**Figure 4:**
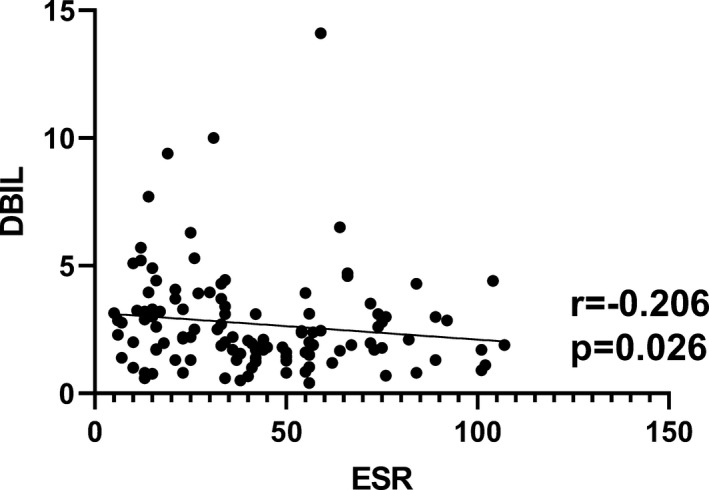
Correlation analysis between ESR and DBIL

As an indicator of inflammation, ESR is one of the important experimental indexes in the evaluation of disease activity. The correlation analyses showed that TBIL, DBIL, and IBIL were negatively correlated with ESR with *r* = −.406, *P* < .001; *r* = −.206, *P* = .026; *r* = −.429, *P* < .001, respectively (Figures 2, 3, 4).

Multiple linear regression analysis was utilized to determine when adjusted for sex, age, ALT, and AST which can affect the levels of bilirubin, whether TBIL, DBIL, and IBIL were independently related to ESR in patients with pSS. Interestingly, our study revealed that TBIL, DBIL, and IBIL were independently correlated with ESR in patients with pSS (*P* < .001, *P* = .048 and *P* < .001) (Tables 2, 3, 4).

**Table 2:** Multiple linear regression analysis between ESR and serum TBIL level in pSS patients

	Unstandardized coefficient		Standardized coefficient	*t*	*P*‐value
	*β*	Standard error	Beta		
Constant	26.161	17.501		1.495	.138
Sex	5.574	7.160	0.069	0.778	.438
Age	0.439	0.149	0.264	2.948	.004
ALT	0.085	0.170	0.125	0.502	.617
AST	−0.048	0.115	−0.103	−0.415	.679
TBIL	−1.988	0.477	−0.361	−4.166	<.001

**Table 3:** Multiple linear regression analysis between ESR and serum DBIL level in pSS patients

	Unstandardized coefficient		Standardized coefficient	*t*	*P*‐value
	*β*	Standard error	Beta		
Constant	13.968	18.153		0.769	.443
Sex	5.554	7.587	0.068	0.732	.466
Age	0.487	0.160	0.294	3.052	.003
ALT	0.211	0.181	0.309	1.167	.246
AST	−0.123	0.121	−0.264	−1.012	.314
DBIL	−2.457	1.227	−0.190	−2.002	.048

**Table 4:** Multiple linear regression analysis between ESR and serum IBIL level in pSS patients

	Unstandardized coefficient		Standardized coefficient	*t*	*P*‐value
	*β*	Standard error	Beta		
Constant	27.694	17.298		1.601	.112
Sex	6.482	7.067	0.080	0.917	.361
Age	0.378	0.148	0.227	2.559	.012
ALT	−0.009	0.171	−0.014	−0.055	.956
AST	−0.003	0.115	−0.006	−0.022	.982
IBIL	−2.894	0.639	−0.393	−4.532	<.001

Abbreviations: ALT, alanine aminotransferase; AST, aspartate aminotransferase; DBIL, direct bilirubin; ESR, erythrocyte sedimentation rate; IBIL, indirect bilirubin; pSS, primary Sjogren's syndrome; TBIL, total bilirubin.

## 4 DISCUSSION

As far as we know, we are the first to report the relationship between the concentration of bilirubin and patients with pSS. We discovered that the concentration of bilirubin was significantly lower in patients with pSS than healthy individuals. Furthermore, our results showed that the levels of bilirubin have high diagnostic value in pSS and were negatively correlated with ESR.

Oxidative stress (OS) is caused by the excessive production of highly active molecules such as reactive nitrogen species (RNS) and reactive oxygen species (ROS), the degree of oxidation exceeds the scavenging of oxides, and the oxidation system and the antioxidant system are out of balance and lead to tissue damage,^16^ which is one of the mechanisms of various diseases such as SLE, RA, and pSS.^17^, ^18^, ^19^ Bilirubin is a type of tetra pyrrole pigment, which mainly comes from the degradation of hemoglobin and the reticular endothelial cells of bone marrow, spleen, and liver. As early as in 1987, Stocker et al revealed that serum bilirubin was a kind of natural antioxidant which can effectively eliminate superoxide and peroxide free radicals and involved in the process of oxidative stress. In a previous study, the levels of serum bilirubin in patients with SLE were significantly lower than healthy control and had important antioxidant and anti‐inflammatory effects in patients with SLE.^12^ Furthermore, You‐Fan Peng et al demonstrated that the concentrations of bilirubin in patients with RA were reduced and assessed the degree of inflammation in patients with RA.^13^ Interestingly, levels of bilirubin in PM patients were significantly reduced when compared with healthy individuals; moreover, they were negatively related with ESR.^14^ These previous researches revealed that the concentrations of serum bilirubin may play a protective role in inflammation‐linked diseases and autoimmune diseases but the diagnosis of bilirubin in patients is lack of specificity.

Indeed, Inflammation is an important feature of primary Sjögren syndrome.^1^ Quite a few studies have demonstrated that immune abnormalities of pSS result in T‐cell and B‐cell abnormalities in the animal model and the patients’ peripheral blood.^1^ Inflammatory cytokines such as interleukin‐17 (IL‐17), interferon‐γ, interleukin‐10 (IL‐10), and interleukin‐37 (IL‐37), which can invade the target organ and promote the occurrence and development of pSS, were produced by aberrant T cells and B cells.^2^ Moreover, ESR, as an indicator of inflammatory response and the disease activity,^20^ has also been observed to be associated with pSS. In our study, we found that TBIL, DBIL, and IBIL were negatively correlated with ESR. These results suggested that bilirubin may play a protective role by inhibiting the production of inflammatory cytokines in pSS. Because of its strong anti‐inflammatory effect, the decrease of serum bilirubin level in patients with pSS may be caused by the excessive consumption of bilirubin by inflammatory reaction. Moreover, with the development of pSS, serum bilirubin was decreased with the excessive consumption of inflammatory activity.

All in all, our study demonstrated that the levels of bilirubin were reduced in patients with pSS and negatively related to ESR. Furthermore, the levels of bilirubin have high diagnostic value in primary Sjögren syndrome. Interestingly, we found that TBIL, DBIL, and IBIL were independently associated with ESR in patients with pSS. Therefore, we speculate that the decrease of serum bilirubin in patients with pSS was caused by inflammation.

However, there are numerous shortcomings in this study. Firstly, this is a retrospective analysis and the sample size is relatively small. Secondly, analysis of the correlation between bilirubin and other inflammatory cytokines such as IL‐17, interferon‐γ, IL‐10, and IL‐37 were not made. Lastly, comparison of the concentration of bilirubin before and after treatment in patients with pSS is required for further observation.
